# Chemical reaction mechanisms in solution from brute force computational Arrhenius plots

**DOI:** 10.1038/ncomms8293

**Published:** 2015-06-01

**Authors:** Masoud Kazemi, Johan Åqvist

**Affiliations:** 1 Department of Cell and Molecular Biology, Uppsala University, Biomedical Center, Box 596, SE-751 24 Uppsala, Sweden

## Abstract

Decomposition of activation free energies of chemical reactions, into enthalpic and entropic components, can provide invaluable signatures of mechanistic pathways both in solution and in enzymes. Owing to the large number of degrees of freedom involved in such condensed-phase reactions, the extensive configurational sampling needed for reliable entropy estimates is still beyond the scope of quantum chemical calculations. Here we show, for the hydrolytic deamination of cytidine and dihydrocytidine in water, how direct computer simulations of the temperature dependence of free energy profiles can be used to extract very accurate thermodynamic activation parameters. The simulations are based on empirical valence bond models, and we demonstrate that the energetics obtained is insensitive to whether these are calibrated by quantum mechanical calculations or experimental data. The thermodynamic activation parameters are in remarkable agreement with experiment results and allow discrimination among alternative mechanisms, as well as rationalization of their different activation enthalpies and entropies.

## Abstract (web summary)

Obtaining activation entropies and enthalpies of a reaction is important for distinguishing between alternative reaction mechanisms. Here the authors use computational methods to accurately obtain the enthalpic/entropic components of the activation free energy for hydrolytic deamination reactions.

## Graphical abstract

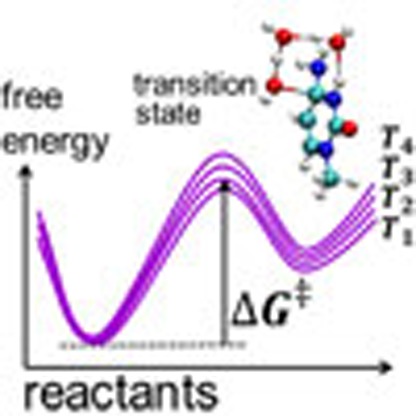

The existence of an entropy of activation for chemical reactions is inherent in transition state (TS) theory, where the activated complex is assumed to be in thermodynamic equilibrium with the reactants. For solution reactions where the transmission coefficient can be assumed to be close to unity, this entropy of activation is typically obtained from experimental Arrhenius plots of the logarithm of the rate against inverse temperature. If activation entropies could be reliably predicted theoretically, then such calculations would be very useful for distinguishing between alternative TS structures of similar energy^1^. However, due to the huge number of degrees of freedom involved in solution reaction dynamics, the extensive configurational sampling required to rigorously obtain activation entropies is presently beyond the scope of quantum chemical calculations. While ideal gas rigid-rotor and harmonic oscillator approximations, in combination with parametrized continuum solvent models, are useful for obtaining thermally corrected activation free energy estimates from quantum mechanical calculations, they do not usually provide sufficiently accurate descriptions of entropic effects. Here we explore a combined method where TS structures and energies are obtained from density functional theory (DFT) calculations, which can then be used to parametrize empirical valence bond (EVB) models^2^,^3^ that allow very extensive all-atom sampling of the reacting system in aqueous solution. This method is used to obtain computational Arrhenius plots for the hydrolytic deamination of cytidine and dihydrocytidine, thereby allowing for direct comparisons with experimental thermodynamic activation parameters.

The spontaneous deamination reaction of cytidine to uridine is of major interest due to its importance for genome instabilities, as cytosine is known to be the nucleic acid base that is most susceptible to hydrolytic deamination^4^. The actual reaction mechanism and energetics of the uncatalysed deamination via attack of a water molecule is also highly relevant for assessing the catalytic power of the enzyme cytidine deaminase. This enzyme, which produces uridine and ammonia from cytidine, has been taken as a prototypic example of an enzyme that achieves its catalytic effect primarily by reducing the loss in activation entropy^5^,^6^. That is, the spontaneous deamination reaction in aqueous solution has been shown to proceed with a large entropy loss of *T*Δ*S*^‡^=−8.3 kcal mol^−1^ at room temperature. The corresponding value for the enzyme-catalysed reaction is *T*Δ*S*^‡^=+0.9 kcal mol^−1^, obtained from the temperature dependence of *k*_cat_^5^. Since the entropy effect on substrate binding (*T*Δ*S*^‡^=−7.6 kcal mol^−1^, derived from *K*_m_) closely matches the difference in activation entropy this may appear as an example of Jencks' so-called ‘Circe effect'^7^, which is by many enzymologists considered to be the main explanation of enzyme catalysis. This hypothesis focuses on the substrate configurational entropy, and essentially states that if it is significantly reduced on binding, that could eliminate the entropy loss associated with reaching the TS.

The overall activation free energy for the uncatalysed deamination of cytidine is 30.4 kcal mol^−1^ at room temperature^5^, corresponding to a rate of about 3 × 10^−10^ s^−1^, which is close to the observed rate for spontaneous cytosine deamination per site of single-stranded DNA^8^. Several theoretical studies have addressed the reaction mechanism of cytidine deamination and proposed that formation of a tetrahedral intermediate is the rate-limiting step of the reaction^9^,^10^,^11^,^12^. Almatarneh *et al.* examined the gas-phase reaction of cytosine with water by quantum mechanical calculations, which showed very large energy barriers that can hardly be relevant for the solution reaction^9^. They also explored the reaction pathway for deamination by hydroxide ion, which resulted in a negatively charged cytosine as the reactant state, lying some 65 kcal mol^−1^ below the Cyt+H_2_O+OH^−^ starting point^10^. Despite the fact that the predicted activation barrier from the Cyt^−^ reactant state was close to the experimentally observed value, this reactant state would not be accessible for the reaction at physiological pH. For the attack by neutral water, Matsubara *et al.* obtained similar results from their density functional calculations, and also found that an auxiliary water molecule could reduce the potential energy barrier to about 40 kcal mol^−1^ in the gas phase^11^. Similar results were obtained in a recent work^12^, which also reported calculations with continuum solvent models. There, the free energy barriers in solution were predicted to be about 35 kcal mol^−1^ for cytosine and 31–33 kcal mol^−1^ for 5,6-dihydrocytosine.

Here we analyse the mechanism and energetics of cytidine and 5,6-dihydrocytidine deamination in water using M06-2X/6-311++G(d,p) DFT calculations^13^ with the SMD continuum solvent model^14^, which together with experimental data^5^,^6^ serve as an input for extensive EVB simulations^2^,^3^. The key point with using the latter method is that it can be unambiguously parametrized for different mechanistic pathways and then used for extensive molecular dynamics (MD) sampling and free energy calculations. This allows us for the first time to accurately obtain the temperature dependence of the activation free energy for a solution reaction directly from computer simulations, and thereby decompose the activation barrier into its enthalpic and entropic components. The thermodynamic decomposition of the free energy barrier is not only in excellent agreement with experimental results, but it also allows us to determine the operational mechanism of cytidine deamination. It thus turns out that, both for cytidine and 5,6-dihydrocytidine, the only pathway compatible with all experimental activation parameters is concerted and involves three water molecules in an eight-membered TS.

## Results

### Stepwise mechanisms

**Figure 1:**
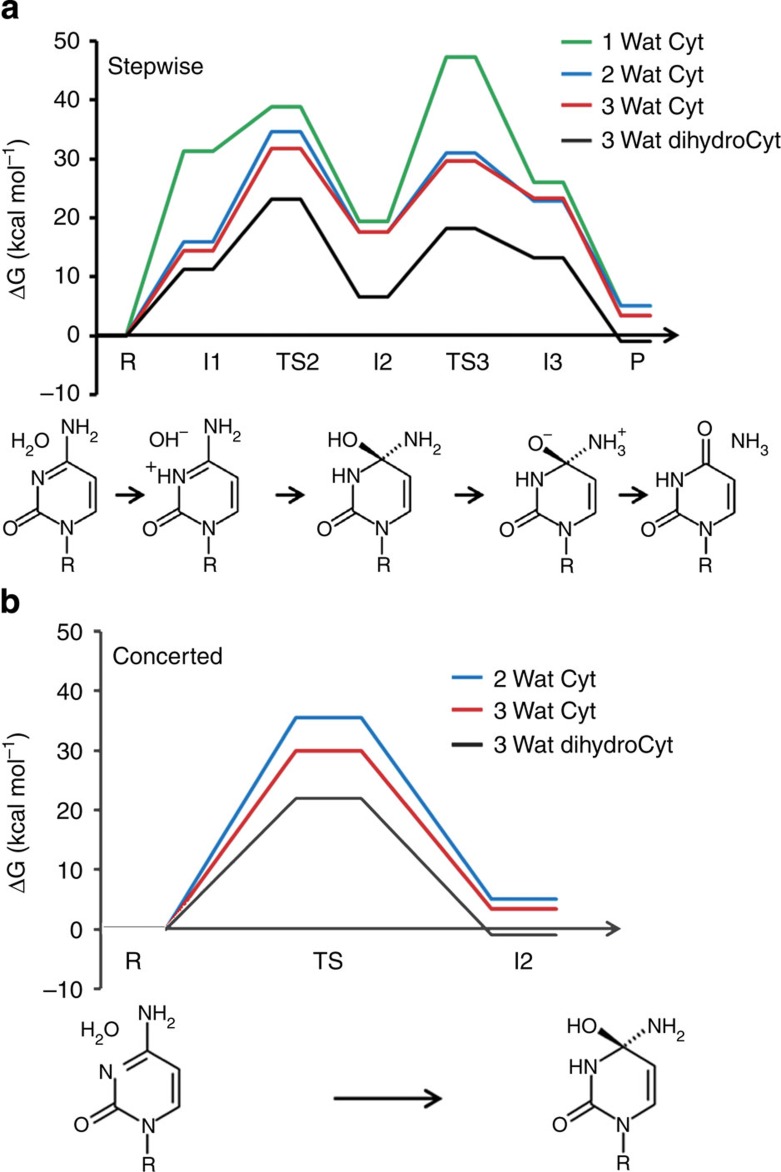
Energetics of hydrolytic deamination of cytidine and 5,6-dihydrocytidine. Calculated free energy profiles (kcal mol^−1^) in aqueous solution for the stepwise (**a**) and concerted (**b**) reaction pathways with the two substrates at the M06-2X/6-311++G**(SMD) level of theory, in the presence of one or two additional explicit water molecules. These do not directly participate in the first half of the stepwise reactions, but mediate PT from the nucleophile to the N3 nitrogen in the concerted cases. The valence bond structures used in subsequent EVB simulations are also shown.

It is clear from the DFT calculations with the SMD solvent model that the deamination of cytidine at neutral pH occurs via the formation of a transient tetrahedral intermediate, resulting from water attack and protonation of the N3 nitrogen. This intermediate is then subsequently deaminated to yield uridine and ammonia (Fig. 1). Formation of the tetrahedral intermediate could go either via a stepwise or a concerted pathway. In the former case, cytidine is protonated at N3 by a water molecule and the resulting hydroxide ion then attacks C4. The energy profile for this two-step pathway was calculated with and without one or two auxiliary water molecules, which do not directly participate in this half of the reaction, but rather act as screening waters. The reaction free energy for the proton transfer (PT) step is predicted to be 15.9 and 14.4 kcal mol^−1^ with two and three water molecules, respectively (Fig. 1a, Supplementary Table 1). Both of these values are very close to the experimental reaction free energy of Δ*G*^0^=15.1 kcal mol^−1^ (at 298 K) that can be estimated from the p*K*_a_ values of water (15.7) and 1-methylcytosine (4.6)^15^. The fact that the three-water case appears more favourable is likely due to improved solvation energy of the protonated cytidine-hydroxide ion complex^16^. It can also be noted that the fact that our calculated PT reaction free energies agree well with the corresponding estimate from bulk p*K*_a_ values indicate that the entropic cost of bringing the resulting hydroxide ion and protonated cytidine into contact, from a 1 M standard state, is counterbalanced by the favourable electrostatic interaction.

**Figure 2:**
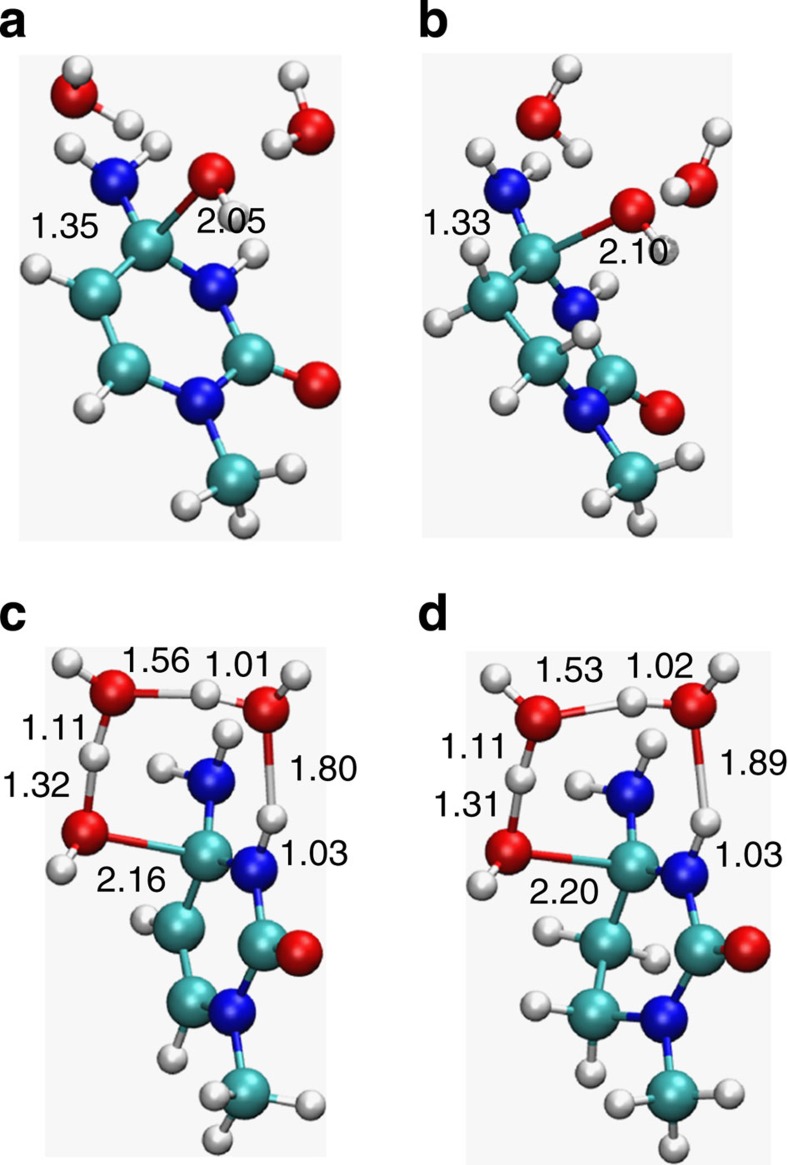
TSs for different possible reaction mechanisms. Optimized rate-limiting transition structures for the stepwise deamination pathways of cytidine (**a**) and 5,6-dihydrocytidine (**b**) and for the corresponding concerted pathways (**c**,**d**), respectively (the two substrates are capped by methyl groups at N1). The lowest-energy TSs with a total of three explicit water molecules are shown and relevant bond distances are given in Å.

The second step involves nucleophilic attack by hydroxide on the C4 carbon, and the TS for this process was also optimized with and without screening waters (Fig. 1a). Similar to the protonation step, the three-water system gives the lowest energy estimate (17.4 kcal mol^−1^) for nucleophilic attack at C4, while the two-water case gives a somewhat higher barrier. Overall, the calculations with three water molecules yield a rate-limiting barrier of 31.8 kcal mol^−1^ for the formation of the tetrahedral intermediate, which is in excellent agreement with the experimentally derived barrier of 30.4 kcal mol^−1^ (ref. 5). The corresponding optimized rate-limiting TS is shown in Fig. 2 and stationary points for the entire reaction are shown in Supplementary Fig. 1. The auxiliary waters thus do not assist directly in any PTs before protonation of the leaving ammonia group in the last TS (Supplementary Fig. 1).

Since the three-water model closely reproduces the available experimental data for cytidine deamination, this model was also used to examine the energetics of the 5,6-dihydrocytidine reaction. The stepwise reaction path resulted in PT to N3 being 11.3 kcal mol^−1^ uphill and a subsequent barrier for the nucleophilic attack of 11.9 kcal mol^−1^ (Fig. 1a, Supplementary Table 1). This yields an overall rate-limiting barrier of 23.2 kcal mol^−1^, which is again in excellent agreement with the experimental barrier of 23.5 kcal mol^−1^ (ref. 6). Furthermore, the optimized TSs for the nucleophilic attack (Fig. 2) are very similar for the two reactants, the C–O bond being marginally longer for 5,6-dihydrocytidine. For both substrates, following the intrinsic reaction coordinate path from TS3 resulted in a local minimum (Supplementary Figs 1 and 2) in which the ammonia group is still bonded to the heterocyclic ring. This intermediate is most stable in the case of 5,6-dihydrocytidine with an activation free energy of 3.3 kcal mol^−1^ for its decomposition (data not shown).

The above results show that the three-water model gives a very good approximation to the activation free energy for both substrates with the stepwise water attack mechanism. Another possible stepwise pathway would be the attack of hydroxide ion on the unprotonated cytidine, which was also examined (data not shown). The predicted activation free energy for such an attack in the presence of two screening waters is 22.8 kcal mol^−1^. This value is, in fact, also in excellent agreement with the corresponding experimental barrier for OH^−^ catalysed deamination of cytidine (24 kcal mol^−1^ at 85 °C and a 55 M standard state, corresponding to the van der Waals contact complex)^17^. However, since the energetic cost of forming a hydroxide ion at pH 7 is about 12 kcal mol^−1^, the overall activation barrier for this type of mechanism appears too high to be compatible with the experimental data. Comparison of the experimental deamination rates at neutral pH and with 1 M KOH also allowed the direct hydroxide mechanism to be ruled out earlier^17^.

### Concerted mechanisms

If the tetrahedral intermediate is formed in a concerted reaction, the protonation at N3 and nucleophilic attack at C4 occur essentially simultaneously. With just a single water molecule (four-membered TS), such a process would involve considerable strain, as was shown by Matsubara *et al.*^11^ who obtained a barrier of 58.6 kcal mol^−1^ in vacuum. These authors, however, also demonstrated a significant barrier reduction down to 39.6 kcal mol^−1^ when a second water participates in the PT chain (six-membered TS). At the M06-2X/6-311++G(d,p)/SMD level of theory, including continuum solvation, we obtain an activation free energy of 35.5 kcal mol^−1^ for the same system (Fig. 1b), with a very similar TS (not shown). Further relaxation of strain is achieved by a three-water mechanism, corresponding to an eight-membered TS (Fig. 2), for which the activation barrier becomes 29.9 kcal mol^−1^ (Fig. 1b). Just as for the stepwise mechanism with three waters, this value is extraordinarily close to the experimentally derived value of 30.4 kcal mol^−1^. The same goes for the concerted reaction with 5,6-dihydrocytidine, where the predicted barrier for the eight-membered TS (Fig. 2) is 22.0 kcal mol^−1^, while the experimental value is 23.5 kcal mol^−1^ (ref. 6). It can be noted here that the solution free energy barriers predicted in ref. 12 with three water molecules (∼35 kcal mol^−1^ for cytosine and 31–33 kcal mol^−1^ for 5,6-dihydrocytosine), at the B3LYP/6-31G(d,p) level of theory with PCM and SMD, correspond to a different type of TS (six-membered) where the third water molecule is dangling rather than participating in an eight-membered TS.

Taken together, our results show that the stepwise and concerted mechanisms have very similar activation energies and that the formation of the tetrahedral intermediate is indeed rate limiting. This also holds for both of the examined substrates and the predicted activation free energies are in good agreement with the available experimental data. However, the experimental Arrhenius plots for spontaneous deamination of cytidine and 5,6-dihydrocytidine give additional and highly interesting information with regard to the reaction energetics^5^,^6^. That is, in the case of cytidine, the entropy contribution (−*T*Δ*S*^‡^=8.3 kcal mol^−1^) to the activation free energy at 25 °C is about one-third of the corresponding enthalpy contribution (Δ*H*^‡^=22.1 kcal mol^−1^). For 5,6-dihydrocytidine, on the other hand, the corresponding values are −*T*Δ*S*^‡^=10.1 and Δ*H*^‡^=13.4 kcal mol^−1^. The activation enthalpy is thus considerably smaller than for cytidine and the entropic contribution to the free energy barrier is now almost as large.

### Arrhenius plots from EVB simulations

To examine to what extent the different alternative deamination mechanisms can explain the above activation enthalpy–entropy partitioning, we turned to MD/EVB simulations of the chemical reactions. It is necessary here to be able to obtain enthalpy and entropy changes for the entire solute–solvent system, to make the connection to the experiment. Without resorting to simplified approximations for the solutes and solvent, as discussed above, the only way is to carry out very extensive configurational sampling of a fully microscopic system. The EVB model^2^,^3^ is ideally suited for this purpose since the reaction surface is parametrized by mixing the key valence bond structures describing the reaction (Fig. 1). Each of these is represented by an analytical force field, which makes the calculations very fast, and allows convergent free energy profiles to be obtained along the relevant reaction paths (for the two reactions considered here, the reported results correspond to over 3 μs of MD simulation). The free energy profiles are obtained using a standard umbrella sampling technique^2^,^3^,^18^. Since we have reliable and similar free energies for the rate-limiting step of the reaction, both from experiments and the DFT calculations, it is straightforward to calibrate EVB potentials that exactly reproduce the desired activation free energies. By running multiple reaction simulations with such a model at different temperatures, computational Arrhenius plots can be constructed and the activation enthalpies and entropies obtained from the relation


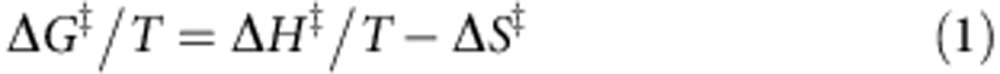


by plotting Δ*G*^‡^/*T* versus 1/*T*.

**Figure 3:**
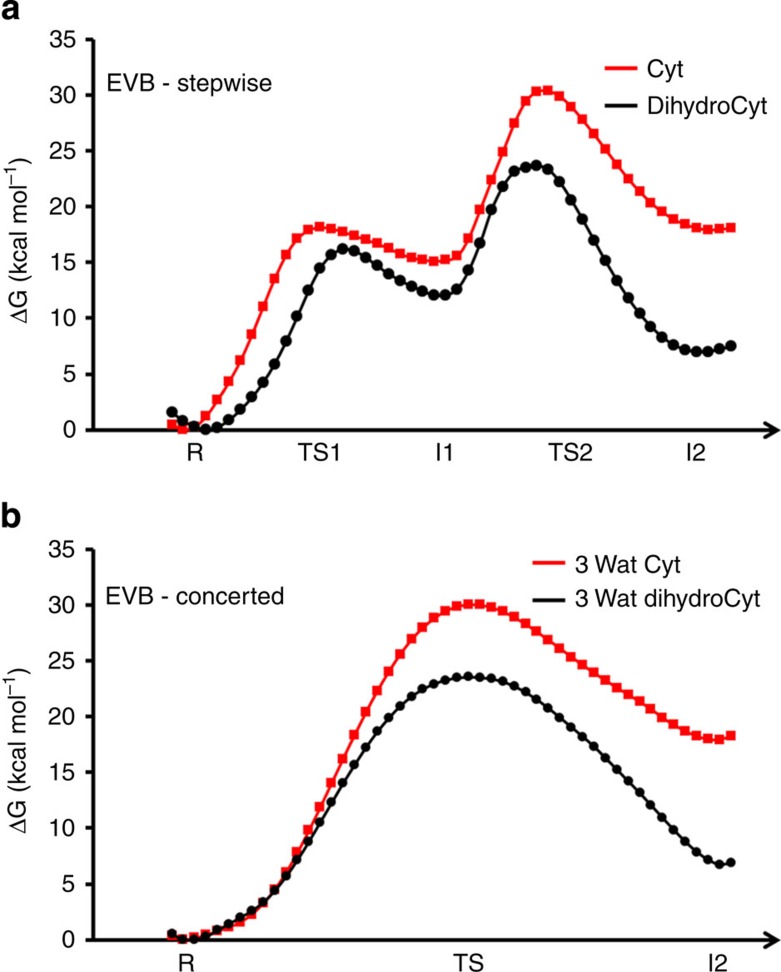
Free energy profiles from EVB simulations. Calculated free energy profiles (kcal mol^−1^) at 298 K from MD/EVB simulations of the stepwise (**a**) and concerted (**b**) pathways for the rate-limiting part of the cytidine and 5,6-dihydrocytidine deamination reactions. The concerted reaction path corresponds to the three-water reaction with an eight-membered TS.

For calibration of the EVB potential surfaces, we can use either the DFT results or the experimental free energy barriers, since they are very similar. Since the former may be associated with larger errors, we find it more unbiased to illustrate the present approach by parametrization against the same experimental data for all relevant possible reaction pathways, that is, the stepwise and the two- and three-water concerted variants. The corresponding results from parametrization directly against the DFT data are given in Supplementary Table 2 and yield exactly the same conclusions. The resulting average EVB reaction free energy profiles at 298 K, each based on 15 independent simulations, are shown in Fig. 3 for the stepwise and three-water concerted pathways for both of the substrates. For the stepwise pathways, the reaction free energy of initial PT step was parametrized from p*K*_a_ values of 4.6 and 6.6 for 1-methylcytosine and 1-methyl-5,6-dihydrocytosine, respectively^15^,^19^. The (non-rate-limiting) intervening activation barriers were taken from Eigen's accurate free energy relationships^20^ as described elsewhere^21^. The overall activation free energies were set to 30.4 and 23.5 kcal mol^−1^ for the two compounds, respectively, in accordance with the experimental results^5^,^6^.

**Figure 4:**
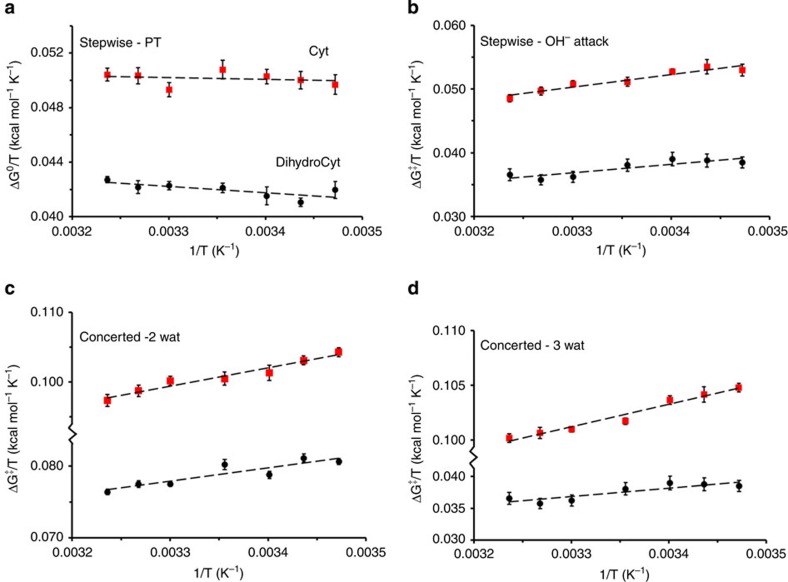
Calculated temperature dependence of different reaction mechanisms. Van't Hoff (**a**) and Arrhenius (**b**) plots from MD/EVB simulations of the stepwise deamination mechanism for cytidine (red squares) and 5,6-dihydrocytidine (black circles). (**c**,**d**) show the corresponding Arrhenius plots for the concerted pathways involving two or three water molecules, respectively. Error bars, 1 s.e.m. from 15 independent simulations.

The EVB simulations give Arrhenius plots with remarkably good fits to straight lines (Fig. 4), which allows the thermodynamic parameters to be extracted with sufficient accuracy. Focusing on the entropic contribution, the overall activation entropies are thus the sum of the reaction entropy for PT from water to the substrate and the activation entropy for nucleophilic attack at C4. The equilibrium constant for the PT reactions show, as expected, only weak temperature dependence as revealed by the van't Hoff plots of Δ*G*^0^/*T* versus 1/*T*, which have small slopes (Fig. 4a). Hence, the PT reaction free energy is dominated by the −*T*Δ*S*^0^ term, which is found to be 16.5 and 17.1 kcal mol^−1^ at 298 K for cytidine and 5,6-dihydrocytidine, respectively (Table 1). The high entropy contribution for this reaction step is mainly due to the ordering of water molecules on going from neutral to zwitterionic reaction species. This behaviour is thus comparable to the ionization of acetic acid in pure water, which proceeds with Δ*G*^0^=6.5 and Δ*H*^0^=−0.1 kcal mol^−1^ (ref. 1). The nucleophilic attack on the protonated substrates, on the other hand, shows a positive activation entropy with *T*Δ*S*^‡^=4.6 and 2.5 kcal mol^−1^ at 298 K for cytidine and 5,6-dihydrocytidine, respectively (Table 1). This favourable contribution can be interpreted such that the solvation effects again dominate, but now the highly localized charges are partially neutralized and the accompanying increase in solvent entropy dominates over the reduction of the configurational space of the reacting groups in the TS.

The EVB simulations for the stepwise mechanisms thus predict the overall activation entropies corresponding to *T*Δ*S*^‡^=−11.9 and −14.6 kcal mol^−1^ (at 298 K) for the two deamination reactions, respectively. Both of these values are about 4 kcal mol^−1^ off from the experimental results, which indicates that the stepwise mechanisms may not properly describe the reactions. The same type of MD/EVB simulations were also carried out for the concerted reaction pathways with both the two- and three-water mechanisms, yielding six- and eight-membered TSs, as discussed above. While the two-water concerted mechanism could probably be excluded already from the DFT results, owing to its significantly higher activation barrier (see above), it is nevertheless instructive to examine its predicted thermodynamic activation parameters. The computed Arrhenius plots for the concerted deamination mechanisms of the two substrates are shown in Fig. 4c,d and the thermodynamic data are summarized in Table 1. There it can be seen that the concerted reaction paths all have significantly less negative activation entropies than the stepwise mechanisms. This basically reflects the avoidance of visiting the zwitterionic state where solvent reorganization imposes a major entropy penalty. It can also be seen that engaging three water molecules (eight-membered TS) in a concerted mechanism, instead of two (six-membered TS), increases the entropic penalty by 3–6 kcal mol^−1^. This energetic cost of ordering an additional water molecule in the TS is, however, counterbalanced by the relieving enthalpic strain in the TS (Table 1).

Comparing the overall thermodynamic data in Table 1, we find that the concerted three-water mechanism is the one that best coincides with the experimental data^5^,^6^. For this mechanism, we see that both the predicted enthalpy and entropy terms are within 1 kcal mol^−1^ of the corresponding experimentally derived values, and for both substrates, which is quite remarkable. It should again perhaps be emphasized that this conclusion is not dependent on our choice of EVB calibration to experimental free energies, since calibration to the DFT results lead to the same conclusion (Supplementary Table 2). It is also noteworthy that the DFT-SMD results for the activation free energies also, in fact, point to this mechanism being slightly more favourable than the competing ones.

## Discussion

To summarize, we have shown how direct computer simulations of the temperature dependence of free energy profiles for chemical reactions in solution can be used to extract reliable thermodynamic activation parameters. It thus appears that this approach is sufficiently robust for making mechanistic predictions and direct comparisons to experiment. A prerequisite is that extensive configurational sampling can be carried out, which was achieved here with the EVB method, but could eventually perhaps be done by other QM/MM methods. The results are also informative with regard to the origin of different activation entropies for alternative mechanisms and highlight the importance of the solvent in this respect. In fact, the underlying reason for why the activation enthalpy–entropy partitioning becomes very precise, although it was in no way built into the EVB models, is that it is mainly determined by the configurational entropy of the solvent, which is correctly captured by the MD sampling. Hence, it is likely that the same approach as used here can also be applied to obtain accurate thermodynamic parameters, via computational van't Hoff plots, for solvation and ligand-binding processes^22^,^23^,^24^.

For the case of spontaneous cytidine deamination, the simulations clearly predict that a concerted eight-membered TS mechanism is at play. A comparison with the enzyme cytidine deaminase, where the same reaction occurs essentially without any entropy loss, further suggests that the origin of this effect may be that hydroxide ion attack dominates the observed activation entropy in that case. Such an explanation would thus be rather different from the view that ‘freezing' substrate motion on binding^7^ is at the heart of favourable enzyme activation entropies.

## Methods

### Quantum mechanical calculations

The different molecular systems explored by DFT calculations consisted of the cytosine and 5,6-hydrocytosine bases, capped by a methyl group at N1, and one, two or three water molecules participating in the hydrolytic reaction. Geometry optimization of all systems was carried out with the hybrid M06-2X hybrid functional^13^ and the 6-311++G(d,p) basis set, using an ultrafine numerical integration grid. The TS structures were validated by frequency calculations at the same level of theory and basis set to confirm stationary points. To verify that the correct minima are connected, intrinsic reaction coordinate calculations were performed for the TSs in both directions. The stepwise mechanisms were optimized with the SMD solvation model^14^ (Supplementary Table 5). The concerted pathways did not yield convergence with SMD and were therefore optimized in the gas phase, with solvation energies calculated at the gas-phase geometries added as corrections to the free energy profiles (Supplementary Table 6). In contrast to the stepwise mechanisms, the charge separation for the concerted pathways is not very significant and, hence, the gas-phase geometries should provide good approximations for evaluating solvation effects^25^. The Gaussian 09 programme^26^ was used for all DFT calculations. All reported DFT energies are free energies obtained from the standard gas-phase thermochemical corrections^26^ in Gaussian 09 plus the SMD solvation free energies. The reactant reference points were the corresponding complexes with water molecules. For 5,6-dihydrocytidine, two different initial conformations were considered in the optimizations, with either C5 or C6 out of plane. In both cases, the final structure converged to the conformation with C6 out of plane and this was used throughout the subsequent calculations.

### EVB simulations

EVB/MD simulations were performed with the programme Q^27^ utilizing spherical boundary conditions, where the full cytidine and 5,6-dihydrocytidine nucleosides were immersed in a TIP3P water droplet of 20 Å radius. The OPLS-AA force field^28^ was used for parametrization of the different valence bond structures via the ffld_server utility in maestro (version 9.2, Schrödinger, LLC, New York, NY, 2011). The non-bonded parameters used in the calculations are given in Supplementary Table 4. Water molecules close to the sphere boundary were subjected to radial and polarization restraints according to the SCAAS model^27^,^29^ and the cytosine ring was restrained to the centre of the sphere with a weak force constant of 0.5 kcal mol^−1^Å^−2^ applied to the C4 atom. Note that, although the difference between the Gibbs' and Helmholtz' free energies is vanishingly small for a solution reaction at normal temperatures and pressures, the SCAAS model formally corresponds more closely to the former as the volume is not strictly constant, but subject to harmonic restraints. The MD simulations were carried out with a 1-fs time step without any nonbonded interaction cutoffs applied to the reacting groups. For water–water interactions (excluding those participating in the reaction), a direct cutoff of 12 Å was applied together with the local reaction field method^30^, which gives an accurate representation of long-range electrostatics.

The valence bond structures used to represent the different reaction pathways are shown in Fig. 1. The stepwise mechanism was simulated via consecutive transformation **R→I1→I2**, while the concerted pathway was represented by the direct transformation **R→I2**. The ground-state EVB free energy profiles Δ*G*(*X*_n_) were calculated as described elsewhere^18^ from


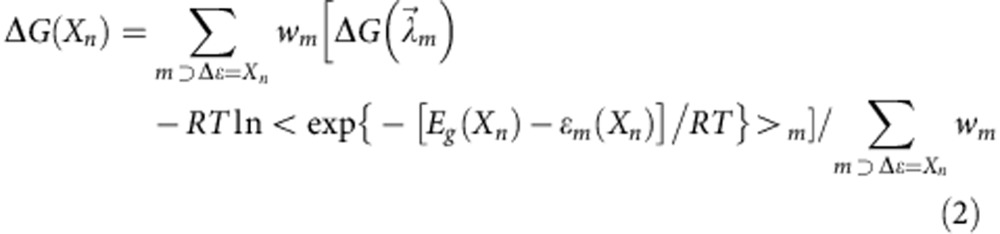


where the discretized reaction coordinate, *X*_*n*_=Δ*ɛ*=*ɛ*_*i*_−*ɛ*_*j*_, is the energy gap between the initial and final diabatic surfaces of the given reaction step. The MD average 〈〉_*m*_ is evaluated on a mapping potential surface *ɛ*_*m*_, given by 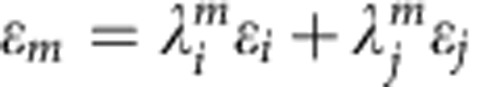, where the mapping vector, 
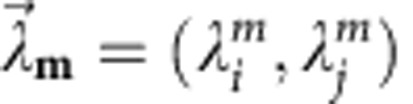
, defines a linear combination between the end-point potentials and changes between the values (1,0) and (0,1). 
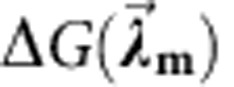
 is the free energy on this mapping potential and is obtained from Zwanzig's exponential formula^31^. *E*_*g*_(*X*_*n*_)is the EVB ground-state energy that is obtained from mixing the diabatic states, via the off-diagonal Hamiltonian matrix elements *H*_*ij*_, and solving the corresponding secular equation^2^,^3^. Finally, different mapping vectors contribute to a given reaction coordinate interval *X*_*n*_ and are weighted proportionally to the total contribution in that interval (
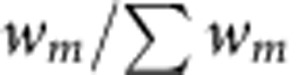
). It may be noted that a major advantage with using the energy gap reaction coordinate together with MD sampling along a linear combination of the end-point potentials is that the system itself is allowed to choose a path of least action, as opposed to imposing geometric constraints to define the reaction path.

The EVB free energy profiles for each reaction step were calculated using 101 different values of 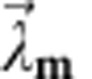, with 50 ps of MD sampling at each 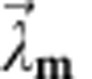. Every such simulation was also repeated 15 times with different initial velocities, yielding about 76 ns of simulation time for each reaction step at each temperature. These simulations were then carried out at seven temperatures from 288 to 309 K to obtain reliable Arrhenius plot of Δ*G*^‡^/*T* versus 1/*T*, for extracting the activation enthalpy and entropy. The stepwise and concerted EVB models for cytidine and 5,6-dihydrocytidine were parametrized at 298 K to either experimental or DFT results by adjusting the relevant *H*_*ij*_ parameters and gas-phase energy shifts^2^,^3^,^18^ (Supplementary Table 3). The barriers for the non-rate-limiting initial PT in the stepwise mechanisms were taken from accurate experimental linear free energy relationships^20^,^21^, as this barrier was difficult to locate in the DFT optimizations (because it is low) and may also be underestimated by that method. Therefore, we consider the experimental estimates^20^,^21^ to be the most reliable.

## Additional information

**How to cite this article:** Masoud Kazemi & Johan Åqvist. Chemical reaction mechanisms in solution from Brute force computational Arrhenius plots. *Nat. Commun.* 6:7293 doi: 10.1038/ncomms8293 (2015).

## Supplementary Material

Supplementary Information Supplementary Figures 1-2 and Supplementary Tables 1-5

## Figures and Tables

**Table 1:** Thermodynamic activation parameters for different mechanisms at 298 K from MD/EVB simulations*.

**Reaction pathway**	**Δ*****G***^**‡**^	**Δ*****H***^**‡**^	***T*****Δ*****S***^**‡**^	**s.e.m.**†
Cyt—proton transfer‡	15.0	−1.5	−16.5	0.18
Cyt—nucleophilic attack	15.3	19.9	4.6	0.21
Cyt—stepwise	30.3	18.4	−11.9	0.28
Cyt—2W concerted	30.3	24.2	−6.1	0.24
Cyt—3W concerted	30.5	21.4	−9.1	0.13
Cyt—experimental	30.4	22.1	−8.3	
dihCyt—proton transfer‡	12.5	−4.6	−17.1	0.13
dihCyt—nucleophilic attack	11.1	13.6	2.5	0.28
dihCyt—stepwise	23.6	9.0	−14.6	0.31
dihCyt—2W concerted	23.5	18.6	−4.9	0.14
diCyt—3W concerted	23.6	12.7	−10.9	0.12
dihCyt—experimental	23.5	13.4	−10.1	

^*^Energies in kcal mol^−1^.

^†^Average s.e.m. for 15 calculated Δ*G*^‡^ values at each of the seven temperatures used to construct Arrhenius (and van't Hoff) plots.

^‡^The overall reaction thermodynamic parameters Δ*G*^0^, Δ*H*^0^ and *T*Δ*S*^0^ are given for the proton transfer step.
