# Supplementary material for: Chemical reaction mechanisms in solution from brute force computational Arrhenius plots
Source: Nat Commun. 2015 Jun 1;6:7293. doi: 10.1038/ncomms8293 (PMC4458863; doi:10.1038/ncomms8293)
Supplement: Supplementary Information — Supplementary Figures 1-2 and Supplementary Tables 1-5 [file ncomms8293-s1.pdf]

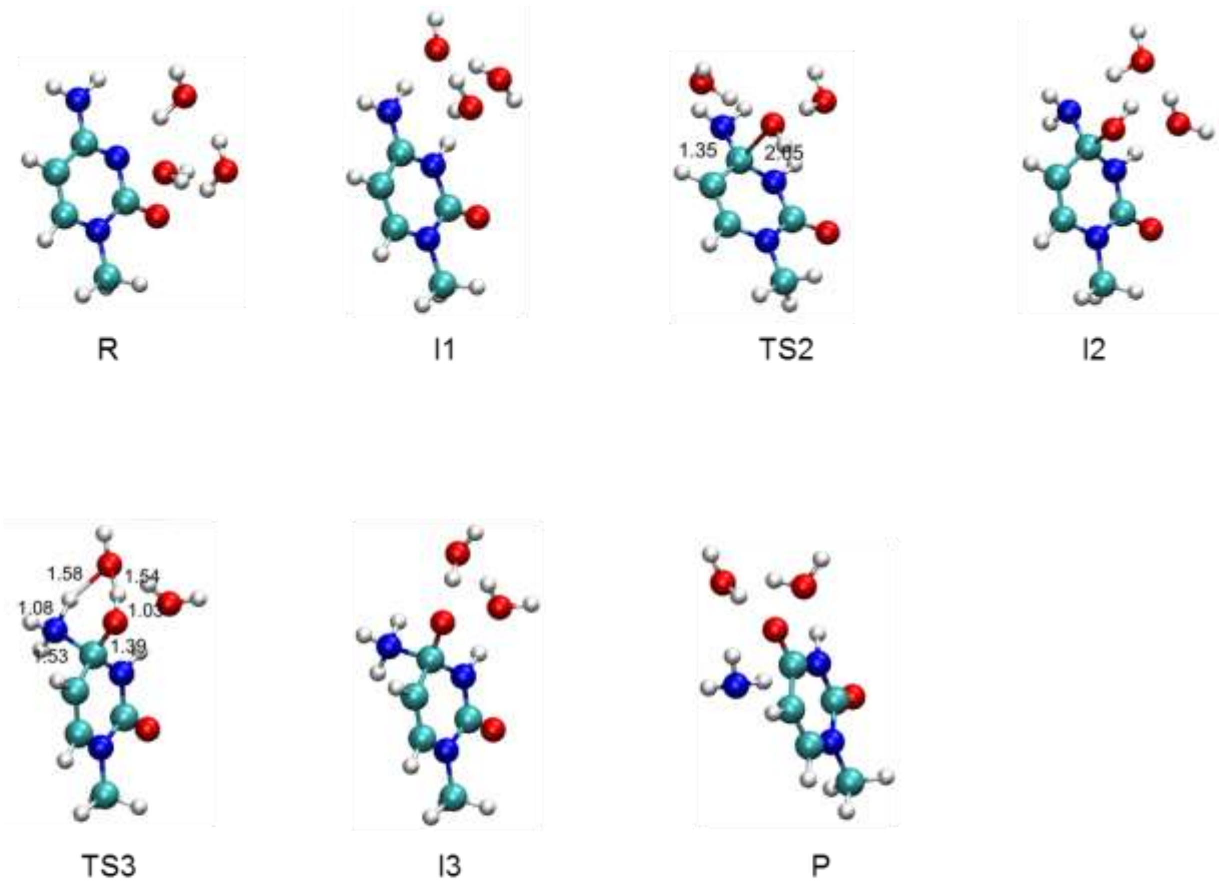

Supplementary Figure 1. Optimized structures for cytidine with two screening waters. Stationary points calculated at the M06-2X/6-311++G**(SMD) level of theory for the stepwise deamination of cytidine (represented here by 1-methylcytosine). The transition states for the initial proton transfer and final ammonia release were not explicitly optimized as their barriers are close in energy to the adjacent intermediates (**I1** and **I3**).

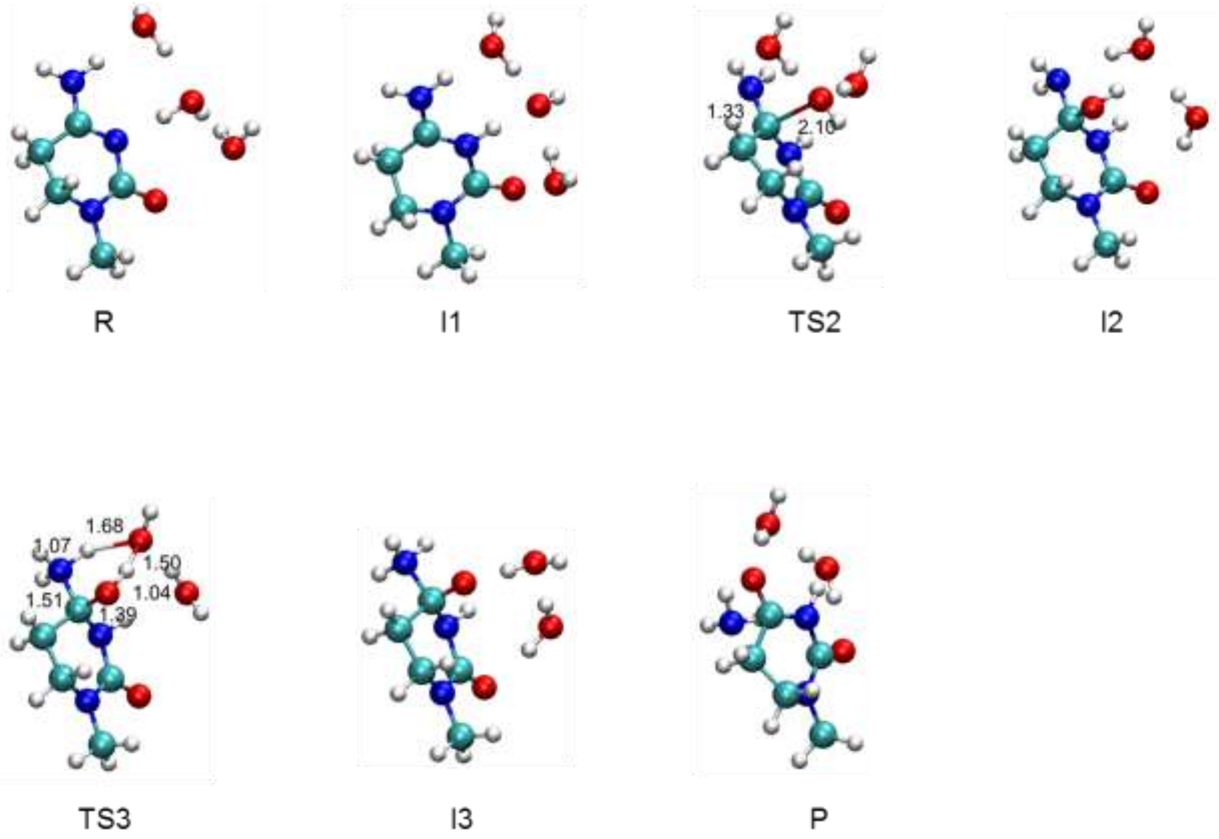

Supplementary Figure 2. Optimized structures for 5,6-dihydrocytidine with two screening waters. Stationary points calculated at the M06-2X/6-311++G**(SMD) level of theory for the stepwise deamination of dihydrocytidine (represented here by 1-methyl-5,6-dihydrocytosine). The transition state for the initial proton transfer was not explicitly optimized and the barrier for final ammonia release was found to be only 3.3 kcal mol^{-1} above **I3**.

Supplementary Table 1. Calculated energetics of different reaction mechanisms at 298K.[a]

Reaction pathway	R	I1	TS2	I2	TS3	I3	P
cytidine – 1W stepwise	0.0	31.3	38.9	19.5	47.3	26.0	5.1
cytidine – 2W stepwise	0.0	15.9	34.6	17.6	30.9	22.9	5.1
cytidine – 3W stepwise	0.0	14.4	31.8	17.6	29.6	23.3	3.4
5,6-dihydrocytidine – 3W stepwise	0.0	11.3	23.2	6.7	18.2	13.2	-0.9
cytidine – 2W concerted	0.0	-	35.5	-	-	-	-
cytidine – 3W concerted	0.0	-	29.9	-	-	-	-
5,6-dihydrocytidine – 3W concerted	0.0	-	22.0	-	-	-	-

[a]Free energies in kcal mol^{-1} from M06-2X/6-311++G**(SMD) calculations. The optimized stationary points correspond to those depicted in Supplementary Figs. 1,2.

Supplementary Table 2. Thermodynamic activation parameters for different mechanisms at 298K from MD/EVB simulations parametrized against the DFT results.[a]

Reaction pathway	$\Delta G^{\ddagger}$	$\Delta H^{\ddagger}$	$T\Delta S^{\ddagger}$	s.e.m.[b]
Cyt - proton transfer[c]	14.4	–2.1	–16.5	0.18
Cyt – nucleophilic attack	17.4	22.8	5.4	0.21
Cyt - stepwise	31.8	20.7	–11.1	0.28
Cyt – 2W concerted	35.5	29.0	–6.5	0.21
Cyt – 3W concerted	29.9	20.1	–9.8	0.12
Cyt - experimental	30.4	22.1	–8.3	
dihCyt – proton transfer[c]	11.4	–5.7	–17.1	0.13
dihCyt – nucleophilic attack	12.0	14.7	2.7	0.28
dihCyt - stepwise	23.4	8.8	–14.6	0.31
diCyt – 3W concerted	22.0	10.8	–11.2	0.12
dihCyt - experimental	23.5	13.4	–10.1	

[a]Energies in kcal mol^{-1}. [b]Average standard error of the mean for calculated $\Delta G^{\ddagger}$ values at the seven temperatures used to construct Arrhenius (and van't Hoff) plots. [c]The overall reaction thermodynamic parameters ΔG^0, ΔH^0 and $T\Delta S^0$ are given for the proton transfer step.

Supplementary Table 3. Target free energy values and EVB parameters used for calibration of different EVB models.[a]

Reaction step	$\Delta G^{\ddagger}_{expt}$	ΔG^{0}_{expt}	$\Delta\alpha$	H_{ij}	$\Delta G^{\ddagger}_{DFT}$	ΔG^{0}_{DFT}	$\Delta\alpha$	H_{ij}
Cyt - proton transfer	18.3[b]	15.1	–85.8	36.0	17.5[b]	14.4	–85.1	36.0
Cyt – nucleophilic attack	15.3	2.5[c]	186.8	63.0	17.4	3.2	183.0	59.0
Cyt – 2W concerted	30.4	17.6[c]	114.5	169.0	35.5	17.6	113.0	150.5
Cyt – 3W concerted	30.4	17.6[c]	112.0	231.5	29.9	17.6	111.5	234.5
dihCyt – proton transfer	16.5[b]	12.4	–136.0	40.0	15.3[b]	11.3	–134.7	40.0
dihCyt – nucleophilic attack	11.1	–5.7[c]	202.0	41.5	11.9	–4.6	200.0	41.5
diCyt – 2W concerted	23.5	6.7[c]	73.0	167.0	–	–	–	–
diCyt – 3W concerted	23.5	6.7[c]	76.0	221.0	22.0	6.7	75.0	231.0

[a]Energies in kcal mol^{-1}. Activation and reaction free energies refer to individual chemical steps. The EVB parameters $\Delta\alpha$ and H_{ij} denote the gas-phase energy shifts and off-diagonal coupling elements between initial and final valence bond states for each reaction step[2,3]. Two different parametrizations are given for each reaction step, corresponding to a fit to either experimental or DFT/SMD results. [b]Proton transfer barrier taken from experimental linear free energy relationships[20,21]. [c]Value taken from the DFT/SMD free energy estimate of the **I2** intermediate, which is not available from experiment.

Supplementary Table 4. Partial atomic charges and van der Waals (Lennard-Jones) parameters for cytidine and 5,6-dihydrocytidine at the stationary points R, I1 and I2 in Fig. 1.

R: cytidine

atom name	partial charge	vdW A_i	vdW B_i
**O1**	-0.6830	760.64	25.04
**C2**	0.1450	944.52	22.03
**C3**	0.1700	944.52	22.03
**O4**	-0.4225	445.13	18.25
**C5**	0.2050	944.52	22.03
**O6**	-0.7000	690.37	23.86
**C7**	0.2050	944.52	22.03
**O8**	-0.7000	690.37	23.86
**C9**	0.4725	1039.88	24.25
**N10**	-0.5600	971.75	28.31
**C11**	0.5500	1802.24	34.18
**O12**	-0.4800	616.44	23.77
**N13**	-0.5400	971.75	28.31
**C14**	0.4600	1039.88	24.25
**N15**	-0.7900	971.75	28.31
**C16**	-0.0600	1039.88	24.25
**C17**	0.1000	1039.88	24.25
**H18**	0.4180	0.00	0.00
**H19**	0.0600	84.57	5.41
**H20**	0.0600	84.57	5.41
**H21**	0.0300	84.57	5.41
**H22**	0.0600	84.57	5.41
**H23**	0.4350	0.00	0.00
**H24**	0.0600	84.57	5.41
**H25**	0.4350	0.00	0.00
**H26**	0.1300	109.18	6.99
**H27**	0.3700	0.00	0.00
**H28**	0.3700	0.00	0.00
**H29**	0.1000	109.18	6.99
**H30**	0.1000	109.18	6.99

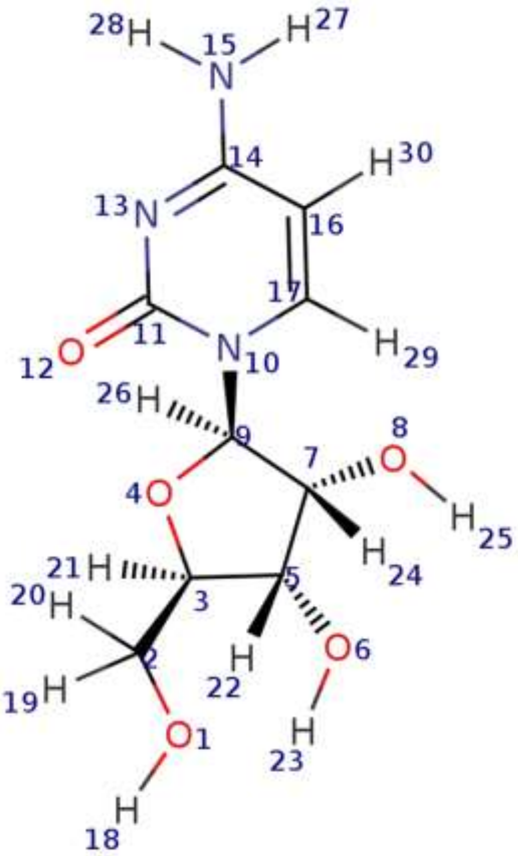

I1: cytidine			
**atom name**	partial charge	vdW A_i	vdW B_i
**O1**	-0.6830	760.64	25.04
**C2**	0.1450	944.52	22.03
**C3**	0.1700	944.52	22.03
**O4**	-0.4225	445.13	18.25
**C5**	0.2050	944.52	22.03
**O6**	-0.7000	690.37	23.86
**C7**	0.2050	944.52	22.03
**O8**	-0.7000	690.37	23.86
**C9**	0.5325	1039.88	24.25
**N10**	-0.6200	971.75	28.31
**C11**	0.6500	1802.24	34.18
**O12**	-0.3000	616.44	23.77
**N13**	-0.7400	971.75	28.31
**C14**	0.6600	1059.13	23.67
**N15**	-0.8100	971.75	28.31
**C16**	-0.0075	1103.59	24.67
**C17**	0.1525	1103.59	24.67
**H18**	0.4180	0.00	0.00
**H19**	0.0600	84.57	5.41
**H20**	0.0600	84.57	5.41
**H21**	0.0300	84.57	5.41
**H22**	0.0600	84.57	5.41
**H23**	0.4350	0.00	0.00
**H24**	0.0600	84.57	5.41
**H25**	0.4350	0.00	0.00
**H26**	0.1600	109.18	6.99
**H27**	0.4600	0.00	0.00
**H28**	0.4300	0.00	0.00
**H29**	0.0875	109.18	6.99
**H30**	0.0875	109.18	6.99
**H31**	0.4800	0.00	0.00

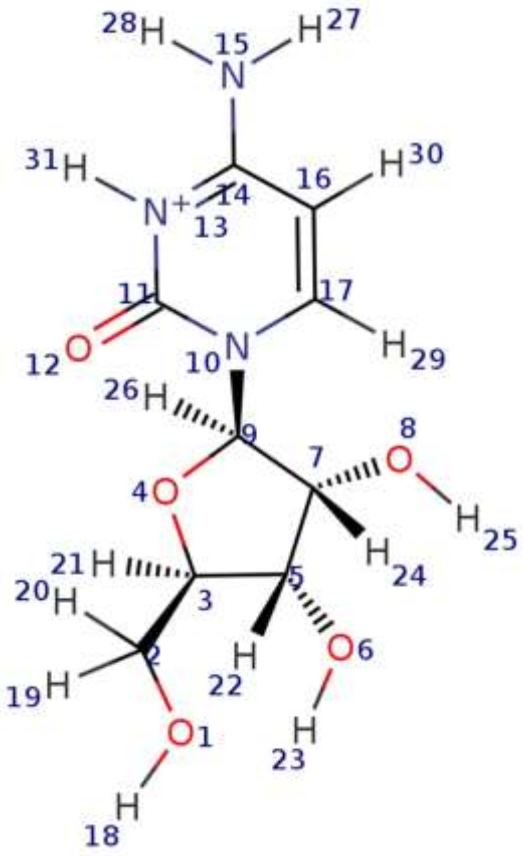

I2: cytidine			
**atom name**	partial charge	vdW A_i	vdW B_i
**O1**	-0.6830	760.64	25.04
**C2**	0.1450	944.52	22.03
**C3**	0.1700	944.52	22.03
**O4**	-0.4000	445.13	18.25
**C5**	0.2050	944.52	22.03
**O6**	-0.7000	690.37	23.86
**C7**	0.2050	944.52	22.03
**O8**	-0.7000	690.37	23.86
**C9**	0.2225	944.52	22.03
**N10**	-0.2450	971.75	28.31
**C11**	0.5000	1802.24	34.18
**O12**	-0.5000	616.44	23.77
**N13**	-0.5000	971.75	28.31
**C14**	0.6450	944.52	22.03
**N15**	-0.9000	1064.97	29.63
**C16**	-0.1150	1103.59	24.67
**C17**	0.0075	1103.59	24.67
**H18**	0.4180	0.00	0.00
**H19**	0.0600	84.57	5.41
**H20**	0.0600	84.57	5.41
**H21**	0.0300	84.57	5.41
**H22**	0.0600	84.57	5.41
**H23**	0.4350	0.00	0.00
**H24**	0.0600	84.57	5.41
**H25**	0.4350	0.00	0.00
**H26**	0.1000	84.57	5.41
**H27**	0.3600	0.00	0.00
**H28**	0.3600	0.00	0.00
**H29**	0.1150	84.57	5.41
**H30**	0.1150	69.58	4.91
**O31**	-0.6830	760.64	25.04
**H32**	0.4180	0.00	0.00
**H33**	0.3000	0.00	0.00

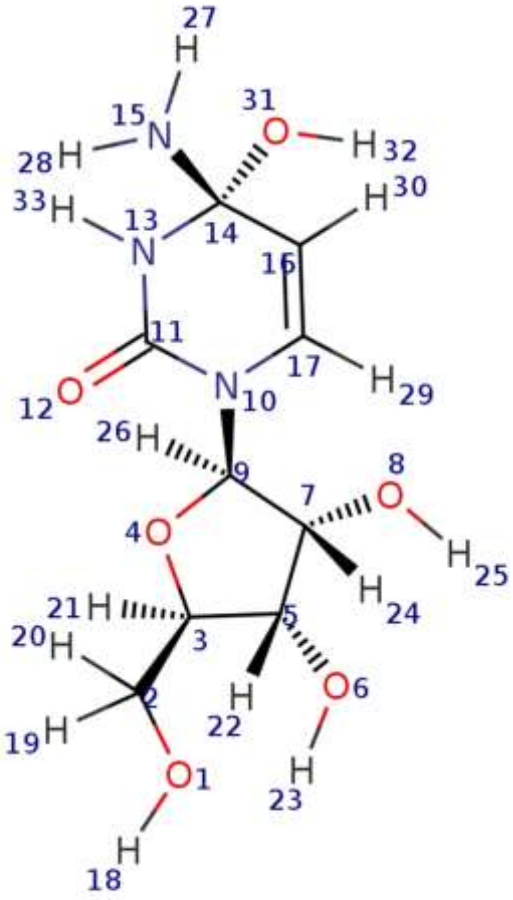

R: 5,6-dihydrocytidine

atom name	partial charge	vdW A_i	vdW B_i
**O1**	-0.6830	760.64	25.04
**C2**	0.1450	944.52	22.03
**C3**	0.1700	944.52	22.03
**O4**	-0.4000	445.13	18.25
**C5**	0.2050	944.52	22.03
**O6**	-0.7000	690.37	23.86
**C7**	0.2050	944.52	22.03
**O8**	-0.7000	690.37	23.86
**C9**	0.2225	944.52	22.03
**N10**	-0.1925	971.75	28.31
**C11**	0.6225	1802.24	34.18
**O12**	-0.5000	616.44	23.77
**N13**	-0.5675	885.43	27.02
**C14**	0.5100	58.02	5.09
**N15**	-0.7850	971.75	28.31
**C16**	-0.1200	944.52	22.03
**C17**	-0.0500	944.52	22.03
**H18**	0.4180	0.00	0.00
**H19**	0.0600	84.57	5.41
**H20**	0.0600	84.57	5.41
**H21**	0.0300	84.57	5.41
**H22**	0.0600	84.57	5.41
**H23**	0.4350	0.00	0.00
**H24**	0.0600	84.57	5.41
**H25**	0.4350	0.00	0.00
**H26**	0.1000	84.57	5.41
**H27**	0.3600	0.00	0.00
**H28**	0.3600	0.00	0.00
**H29**	0.0600	84.57	5.41
**H30**	0.0600	84.57	5.41
**H31**	0.0600	84.57	5.41
**H32**	0.0600	84.57	5.41

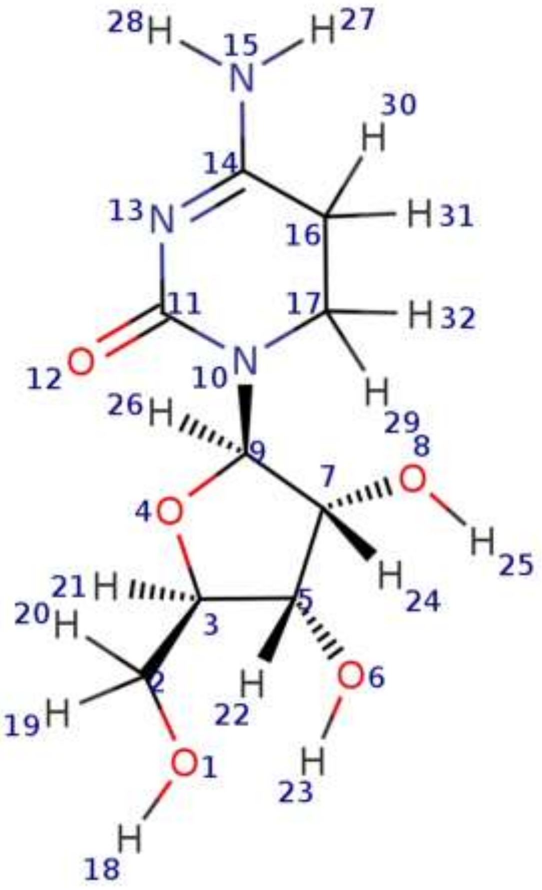

I1: 5,6-dihydrocytidine			
**atom name**	partial charge	vdW A_i	vdW B_i
**O1**	-0.6830	760.64	25.04
**C2**	0.1450	944.52	22.03
**C3**	0.1700	944.52	22.03
**O4**	-0.4000	445.13	18.25
**C5**	0.2050	944.52	22.03
**O6**	-0.7000	690.37	23.86
**C7**	0.2050	944.52	22.03
**O8**	-0.7000	690.37	23.86
**C9**	0.2225	944.52	22.03
**N10**	-0.1925	971.75	28.31
**C11**	0.6225	1802.24	34.18
**O12**	-0.5000	616.44	23.77
**N13**	-0.5805	971.75	28.31
**C14**	0.9840	58.02	5.09
**N15**	-0.9240	971.75	28.31
**C16**	-0.1200	944.52	22.03
**C17**	-0.0500	944.52	22.03
**H18**	0.4180	0.00	0.00
**H19**	0.0600	84.57	5.41
**H20**	0.0600	84.57	5.41
**H21**	0.0300	84.57	5.41
**H22**	0.0600	84.57	5.41
**H23**	0.4350	0.00	0.00
**H24**	0.0600	84.57	5.41
**H25**	0.4350	0.00	0.00
**H26**	0.1000	84.57	5.41
**H27**	0.4660	0.00	0.00
**H28**	0.4660	0.00	0.00
**H29**	0.0600	84.57	5.41
**H30**	0.0600	84.57	5.41
**H31**	0.0600	84.57	5.41
**H32**	0.0600	84.57	5.41
**H33**	0.4660	0.00	0.00

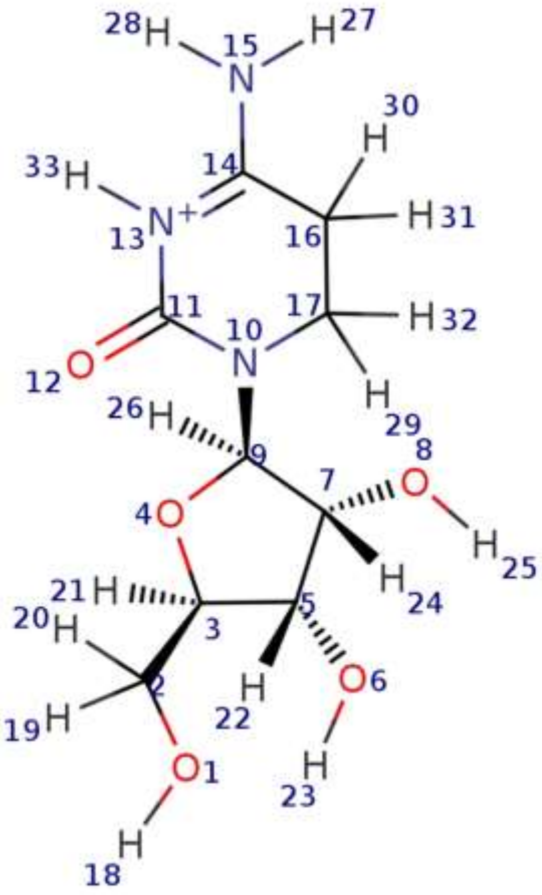

I2: 5,6-dihydrocytidine			
**atom name**	partial charge	vdW A_i	vdW B_i
**O1**	-0.6830	760.64	25.04
**C2**	0.1450	944.52	22.03
**C3**	0.1700	944.52	22.03
**O4**	-0.4000	445.13	18.25
**C5**	0.2050	944.52	22.03
**O6**	-0.7000	690.37	23.86
**C7**	0.2050	944.52	22.03
**O8**	-0.7000	690.37	23.86
**C9**	0.2225	944.52	22.03
**N10**	-0.1925	971.75	28.31
**C11**	0.5000	1802.24	34.18
**O12**	-0.5000	616.44	23.77
**N13**	-0.5000	971.75	28.31
**C14**	0.6450	944.52	22.03
**N15**	-0.9000	1064.97	29.63
**C16**	-0.1200	944.52	22.03
**C17**	-0.0500	944.52	22.03
**H18**	0.4180	0.00	0.00
**H19**	0.0600	84.57	5.41
**H20**	0.0600	84.57	5.41
**H21**	0.0300	84.57	5.41
**H22**	0.0600	84.57	5.41
**H23**	0.4350	0.00	0.00
**H24**	0.0600	84.57	5.41
**H25**	0.4350	0.00	0.00
**H26**	0.1000	84.57	5.41
**H27**	0.3600	0.00	0.00
**H28**	0.3600	0.00	0.00
**H29**	0.0600	84.57	5.41
**H30**	0.0600	84.57	5.41
**O31**	-0.6830	760.64	25.04
**H32**	0.4180	0.00	0.00
**H33**	0.3000	0.00	0.00
**H34**	0.0600	84.57	5.41
**H35**	0.0600	84.57	5.41

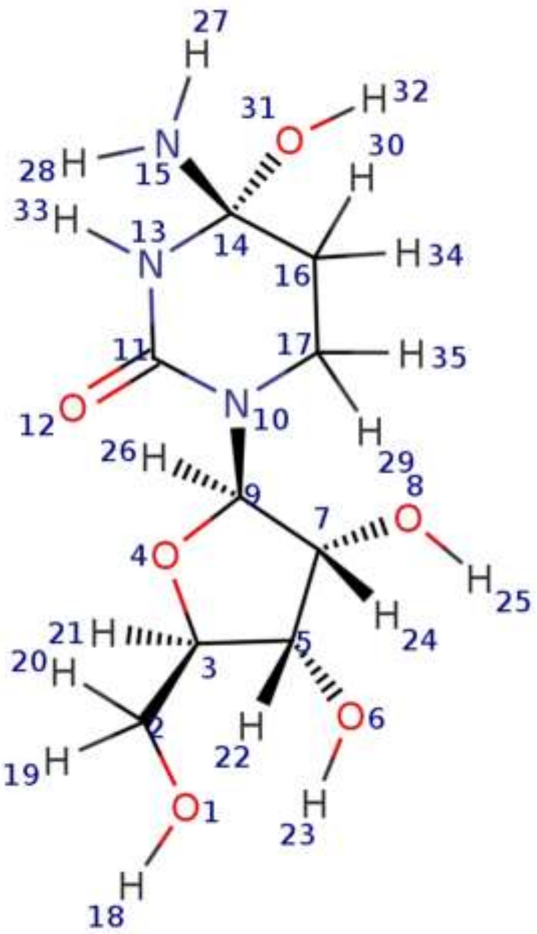

Supplementary Table 5. Optimized geometries and energetics at the M06-2X/6-311++G(SMD) level for the three-water stepwise mechanism.**

R: cytidine

N	-1.795	0.393	0.243
C	-2.689	1.550	0.287
C	-0.500	0.545	0.726
H	-2.815	1.880	1.318
H	-2.271	2.366	-0.302
H	-3.652	1.256	-0.123
C	-2.190	-0.785	-0.307
N	0.335	-0.519	0.719
O	-0.152	1.657	1.161
C	-1.359	-1.850	-0.369
H	-3.204	-0.816	-0.686
C	-0.054	-1.673	0.175
H	2.177	-0.270	0.878
H	-1.667	-2.788	-0.807
N	0.823	-2.683	0.157
H	1.749	-2.556	0.542
H	0.574	-3.572	-0.250
O	3.146	-0.125	0.823
H	3.470	-0.857	0.285
H	2.665	1.447	-0.173
O	2.068	2.120	-0.542
H	1.348	2.157	0.111
H	1.108	1.033	-1.722
O	0.579	0.447	-2.294
H	0.594	0.873	-3.158

Zero-point correction	0.20127
Thermal correction to Energy	0.21833
Thermal correction to Enthalpy	0.21928
Thermal correction to Gibbs Free Energy	0.15725
Sum of electronic and zero-point Energies	-663.35234
Sum of electronic and thermal Energies	-663.33527
Sum of electronic and thermal Enthalpies	-663.33433
Sum of electronic and thermal Free Energies	-663.39635

I1: cytidine

N	2.369	0.064	0.078
C	3.635	0.784	0.248
C	1.232	0.822	-0.091
H	3.801	1.436	-0.608
H	3.601	1.379	1.160
H	4.435	0.052	0.315
C	2.311	-1.298	0.084
N	0.058	0.116	-0.252
O	1.250	2.042	-0.102
C	1.155	-1.981	-0.071
H	3.257	-1.804	0.225
C	-0.042	-1.236	-0.251
H	-0.762	0.698	-0.424
H	1.127	-3.060	-0.061
N	-1.217	-1.797	-0.419
H	-2.082	-1.269	-0.485
H	-1.260	-2.810	-0.402
O	-2.122	1.757	-0.859
H	-2.094	1.971	-1.797
H	-2.776	0.968	-0.759
O	-2.625	0.298	2.007
H	-2.167	1.132	1.857
H	-3.020	0.066	1.121
O	-3.485	-0.301	-0.407
H	-4.418	-0.358	-0.627

Zero-point correction	0.19914
Thermal correction to Energy	0.21555
Thermal correction to Enthalpy	0.21649
Thermal correction to Gibbs Free Energy	0.15391
Sum of electronic and zero-point Energies	-663.32820
Sum of electronic and thermal Energies	-663.31180
Sum of electronic and thermal Enthalpies	-663.31085
Sum of electronic and thermal Free Energies	-663.37344

TS2: cytidine

N	1.984	-0.593	0.071
C	3.335	-0.787	0.603
C	1.150	-1.659	-0.189
H	4.065	-0.345	-0.072
H	3.419	-0.324	1.586
H	3.516	-1.856	0.688
C	1.577	0.696	-0.157
C	-0.117	-1.500	-0.601
H	1.589	-2.635	-0.035
N	0.327	0.836	-0.722
O	2.283	1.667	0.082
C	-0.621	-0.156	-0.748
H	-0.769	-2.340	-0.788
H	-0.008	1.798	-0.774
N	-1.733	0.084	-1.471
H	-2.087	1.035	-1.437
H	-2.451	-0.628	-1.389
O	-3.276	-1.651	0.605
H	-3.642	-2.022	1.413
H	-2.566	-1.013	0.901
O	-1.626	2.645	0.499
H	-2.547	2.793	0.258
H	-1.589	1.707	0.838
O	-1.406	0.104	1.124
H	-0.708	-0.138	1.743

Zero-point correction	0.20082
Thermal correction to Energy	0.21632
Thermal correction to Enthalpy	0.21726
Thermal correction to Gibbs Free Energy	0.15798
Sum of electronic and zero-point Energies	-663.30284
Sum of electronic and thermal Energies	-663.28735
Sum of electronic and thermal Enthalpies	-663.28640
Sum of electronic and thermal Free Energies	-663.34569

I2: cytidine

C	0.403	-1.078	0.012
N	0.097	0.322	0.219
C	-0.850	-1.881	0.163
C	-1.116	0.913	0.049
H	0.883	0.974	0.225
N	1.473	-1.426	0.946
O	0.898	-1.324	-1.295
C	-2.045	-1.294	0.115
H	-0.754	-2.953	0.272
N	-2.201	0.081	-0.032
O	-1.241	2.143	0.010
H	1.666	-2.421	0.840
H	1.139	-1.289	1.897
H	1.778	-0.925	-1.372
H	-2.969	-1.851	0.189
C	-3.529	0.681	-0.131
H	-3.776	1.236	0.776
H	-3.575	1.353	-0.986
H	-4.252	-0.119	-0.271
O	3.591	-0.019	-0.260
H	4.487	-0.278	-0.017
H	2.990	-0.532	0.326
O	2.315	2.376	0.233
H	2.211	2.875	-0.584
H	2.916	1.641	0.009

Zero-point correction	0.20477
Thermal correction to Energy	0.22022
Thermal correction to Enthalpy	0.22117
Thermal correction to Gibbs Free Energy	0.16240
Sum of electronic and zero-point Energies	-663.32597
Sum of electronic and thermal Energies	-663.31052
Sum of electronic and thermal Enthalpies	-663.30958
Sum of electronic and thermal Free Energies	-663.36834

TS3: cytidine

N	-2.194	-0.001	0.046
C	-3.567	0.494	0.140
C	-1.931	-1.338	-0.210
H	-3.668	1.145	1.006
H	-3.844	1.045	-0.761
H	-4.229	-0.361	0.258
C	-1.178	0.920	0.025
C	-0.697	-1.829	-0.328
H	-2.808	-1.965	-0.308
N	0.085	0.435	-0.131
O	-1.396	2.130	0.120
C	0.487	-0.947	-0.149
H	-0.516	-2.877	-0.522
H	0.826	1.142	-0.151
N	1.168	-1.248	1.190
O	1.461	-1.186	-1.111
H	1.290	-2.259	1.289
H	0.606	-0.905	1.970
O	2.405	2.287	-0.143
H	2.619	2.567	-1.039
H	2.886	1.420	-0.010
H	2.328	-0.770	-0.752
H	2.142	-0.779	1.123
O	3.290	-0.115	0.257
H	4.193	-0.442	0.298

Zero-point correction	0.20242
Thermal correction to Energy	0.21633
Thermal correction to Enthalpy	0.21728
Thermal correction to Gibbs Free Energy	0.16154
Sum of electronic and zero-point Energies	-663.30827
Sum of electronic and thermal Energies	-663.29435
Sum of electronic and thermal Enthalpies	-663.29341
Sum of electronic and thermal Free Energies	-663.34915

I3: cytidine

N	-2.214	0.049	-0.037
C	-3.566	0.598	0.046
C	-2.004	-1.307	-0.266
H	-3.652	1.246	0.917
H	-3.814	1.166	-0.851
H	-4.262	-0.231	0.151
C	-1.160	0.928	-0.066
C	-0.789	-1.852	-0.327
H	-2.908	-1.888	-0.399
N	0.077	0.388	-0.227
O	-1.337	2.149	0.012
C	0.438	-1.018	-0.121
H	-0.653	-2.909	-0.512
H	0.849	1.058	-0.193
O	1.506	-1.348	-0.814
N	0.828	-1.209	1.435
H	0.066	-0.940	2.061
H	1.064	-2.192	1.590
H	1.652	-0.637	1.638
O	2.390	2.233	0.158
H	2.553	2.841	-0.570
H	2.999	1.482	0.011
H	2.921	-0.617	-0.439
O	3.749	-0.141	-0.145
H	4.372	-0.218	-0.875

Zero-point correction	0.20413
Thermal correction to Energy	0.21966
Thermal correction to Enthalpy	0.22060
Thermal correction to Gibbs Free Energy	0.16109
Sum of electronic and zero-point Energies	-663.31613
Sum of electronic and thermal Energies	-663.30060
Sum of electronic and thermal Enthalpies	-663.29966
Sum of electronic and thermal Free Energies	-663.35918

P: cytidine

N	-2.134	-0.026	-0.020
C	-3.377	0.702	0.252
C	-2.018	-1.365	0.227
H	-3.222	1.417	1.060
H	-3.696	1.231	-0.645
H	-4.136	-0.019	0.541
C	-1.058	0.705	-0.472
C	-0.872	-2.051	0.033
H	-2.917	-1.845	0.594
N	0.080	-0.015	-0.737
O	-1.116	1.916	-0.643
C	0.288	-1.347	-0.439
H	-0.802	-3.106	0.250
H	0.905	0.555	-0.975
O	1.410	-1.844	-0.594
O	2.394	1.683	-0.921
H	2.872	1.772	-1.752
H	2.929	1.069	-0.383
O	3.468	-0.369	0.620
H	4.353	-0.712	0.453
H	2.860	-0.993	0.186
N	0.727	0.926	2.080
H	1.083	1.279	2.963
H	0.519	1.742	1.510
H	1.506	0.453	1.626

Zero-point correction	0.20134
Thermal correction to Energy	0.21885
Thermal correction to Enthalpy	0.21979
Thermal correction to Gibbs Free Energy	0.15588
Sum of electronic and zero-point Energies	-663.34547
Sum of electronic and thermal Energies	-663.32796
Sum of electronic and thermal Enthalpies	-663.32702
Sum of electronic and thermal Free Energies	-663.39092

R: 5,6-dihydrocytidine

N	2.180	0.155	-0.182
C	3.242	1.153	-0.163
C	2.346	-1.008	0.684
H	4.197	0.637	-0.074
H	3.128	1.841	0.682
H	3.238	1.725	-1.088
C	0.926	0.559	-0.516
C	1.406	-2.104	0.226
H	3.379	-1.342	0.615
H	2.141	-0.736	1.726
N	-0.160	-0.279	-0.293
O	0.722	1.673	-1.021
C	0.033	-1.527	0.067
H	1.392	-2.934	0.933
H	1.716	-2.489	-0.750
H	-1.640	0.634	-0.488
N	-1.007	-2.323	0.271
H	-1.962	-1.987	0.136
H	-0.854	-3.278	0.562
O	-3.750	-1.275	-0.066
H	-4.243	-1.586	-0.833
H	-3.473	-0.368	-0.289
O	-1.413	3.090	1.259
H	-2.103	3.321	1.889
H	-1.835	2.458	0.650
O	-2.480	1.167	-0.503
H	-2.525	1.552	-1.386

Zero-point correction	0.22462
Thermal correction to Energy	0.24219
Thermal correction to Enthalpy	0.24314
Thermal correction to Gibbs Free Energy	0.17864
Sum of electronic and zero-point Energies	-664.52956
Sum of electronic and thermal Energies	-664.51199
Sum of electronic and thermal Enthalpies	-664.51104
Sum of electronic and thermal Free Energies	-664.57554

I1: 5,6-dihydrocytidine

N	2.164	0.067	-0.181
C	3.311	0.969	-0.117
C	2.211	-1.133	0.655
H	4.219	0.369	-0.106
H	3.271	1.585	0.785
H	3.324	1.613	-0.993
C	0.976	0.599	-0.519
C	1.193	-2.143	0.160
H	3.212	-1.555	0.583
H	2.020	-0.868	1.700
N	-0.170	-0.202	-0.319
O	0.819	1.719	-0.991
C	-0.138	-1.489	0.028
H	1.122	-2.995	0.834
H	1.469	-2.506	-0.835
H	-1.047	0.302	-0.429
N	-1.241	-2.157	0.216
H	-2.157	-1.741	0.091
H	-1.174	-3.130	0.489
O	-3.739	-0.871	-0.088
H	-4.240	-1.083	-0.882
H	-3.267	0.024	-0.279
O	-1.099	2.701	1.317
H	-1.746	2.977	1.973
H	-1.623	2.182	0.639
O	-2.411	1.247	-0.430
H	-2.527	1.689	-1.277

Zero-point correction	0.22354
Thermal correction to Energy	0.24027
Thermal correction to Enthalpy	0.24121
Thermal correction to Gibbs Free Energy	0.17927
Sum of electronic and zero-point Energies	-664.51324
Sum of electronic and thermal Energies	-664.49651
Sum of electronic and thermal Enthalpies	-664.49556
Sum of electronic and thermal Free Energies	-664.55750

TS2: 5,6-dihydrocytidine

N	-1.894	-0.642	0.022
C	-3.084	-0.895	-0.786
C	-0.889	-1.711	0.001
H	-3.343	-1.947	-0.686
H	-2.894	-0.670	-1.840
H	-3.915	-0.290	-0.431
C	-1.579	0.646	0.257
C	0.263	-1.423	0.942
H	-1.382	-2.633	0.308
H	-0.533	-1.847	-1.024
N	-0.350	0.911	0.878
O	-2.318	1.596	0.005
C	0.668	0.013	0.902
H	1.118	-2.057	0.713
H	-0.038	-1.607	1.978
H	-0.104	1.898	0.872
N	1.818	0.386	1.447
H	2.066	1.369	1.429
H	2.581	-0.278	1.404
O	1.289	2.669	-0.756
H	1.315	1.692	-0.981
H	2.085	3.046	-1.140
O	3.148	-1.658	-0.743
H	3.879	-1.419	-1.321
H	2.421	-1.001	-0.954
O	1.244	0.084	-1.121
H	0.543	-0.156	-1.736

Zero-point correction	0.22474
Thermal correction to Energy	0.24070
Thermal correction to Enthalpy	0.24165
Thermal correction to Gibbs Free Energy	0.1818
Sum of electronic and zero-point Energies	-664.49574
Sum of electronic and thermal Energies	-664.47978
Sum of electronic and thermal Enthalpies	-664.47883
Sum of electronic and thermal Free Energies	-664.53866

I2: 5,6-dihydrocytidine

C	-0.509	-1.182	-0.056
C	0.743	-2.041	-0.016
N	-1.691	-1.805	-0.627
C	1.914	-1.228	0.508
H	0.571	-2.899	0.634
H	0.939	-2.401	-1.027
N	-0.214	-0.023	-0.877
O	-0.798	-0.820	1.281
H	-1.905	-2.636	-0.078
H	-1.481	-2.115	-1.573
N	1.994	0.097	-0.119
H	2.851	-1.749	0.303
H	1.837	-1.097	1.592
C	0.908	0.736	-0.607
H	-1.019	0.561	-1.084
H	-1.543	-0.200	1.274
C	3.124	0.915	0.307
O	0.906	1.950	-0.877
H	3.986	0.263	0.433
H	2.915	1.416	1.257
H	3.354	1.662	-0.449
O	-3.340	0.482	-0.063
H	-3.987	0.274	0.621
H	-2.977	-0.382	-0.349
O	-1.560	2.612	0.708
H	-0.765	2.525	0.162
H	-2.149	1.893	0.424

Zero-point correction	0.22874
Thermal correction to Energy	0.24503
Thermal correction to Enthalpy	0.24598
Thermal correction to Gibbs Free Energy	0.18528
Sum of electronic and zero-point Energies	-664.52146
Sum of electronic and thermal Energies	-664.50516
Sum of electronic and thermal Enthalpies	-664.50422
Sum of electronic and thermal Free Energies	-664.56491

TS3: 5,6-dihydrocytidine

N	2.102	0.112	-0.040
C	3.321	0.775	0.410
C	1.911	-1.262	0.433
H	4.131	0.047	0.390
H	3.212	1.160	1.429
H	3.569	1.596	-0.258
C	1.050	0.899	-0.371
C	0.740	-1.915	-0.272
H	2.824	-1.819	0.224
H	1.757	-1.260	1.517
N	-0.161	0.288	-0.656
O	1.147	2.127	-0.489
C	-0.472	-1.024	-0.131
H	0.522	-2.883	0.181
H	0.959	-2.057	-1.332
H	-0.925	0.965	-0.617
N	-1.614	-1.540	-0.970
O	-0.927	-1.005	1.180
H	-1.773	-2.528	-0.755
H	-1.425	-1.439	-1.969
O	-2.334	2.454	0.024
H	-1.732	2.705	0.733
H	-2.706	1.563	0.292
H	-1.854	-0.541	1.135
H	-2.465	-0.981	-0.654
O	-3.104	0.024	0.527
H	-3.920	-0.195	0.986

Zero-point correction	0.22769
Thermal correction to Energy	0.24175
Thermal correction to Enthalpy	0.24270
Thermal correction to Gibbs Free Energy	0.18727
Sum of electronic and zero-point Energies	-664.50616
Sum of electronic and thermal Energies	-664.49210
Sum of electronic and thermal Enthalpies	-664.49115
Sum of electronic and thermal Free Energies	-664.54658

I3: 5,6-dihydrocytidine

C	-0.091	-1.418	-0.102
N	-0.736	-2.451	-1.067
C	1.395	-1.750	-0.034
H	-0.670	-3.377	-0.636
H	-1.728	-2.226	-1.181
H	-0.285	-2.472	-1.985
N	-0.219	-0.153	-0.848
O	-0.740	-1.457	1.048
C	2.100	-0.610	0.671
H	1.522	-2.672	0.535
H	1.806	-1.890	-1.036
C	0.584	0.922	-0.527
H	-1.184	0.142	-0.983
N	1.788	0.676	0.039
H	3.180	-0.749	0.623
H	1.812	-0.575	1.727
O	0.215	2.074	-0.819
C	2.550	1.818	0.533
H	3.578	1.496	0.694
H	2.140	2.189	1.477
H	2.545	2.621	-0.201
O	-2.939	-0.021	1.105
H	-3.122	0.170	2.031
H	-2.101	-0.568	1.103
O	-2.567	2.368	-0.324
H	-1.621	2.367	-0.549
H	-2.692	1.548	0.188

Zero-point correction	0.23106
Thermal correction to Energy	0.24578
Thermal correction to Enthalpy	0.24672
Thermal correction to Gibbs Free Energy	0.19034
Sum of electronic and zero-point Energies	-664.51383
Sum of electronic and thermal Energies	-664.49911
Sum of electronic and thermal Enthalpies	-664.49817
Sum of electronic and thermal Free Energies	-664.55454

P: 5,6-dihydrocytidine

N	-1.967	0.396	-0.003
C	-2.985	1.405	0.262
C	-2.374	-1.002	-0.160
H	-3.689	1.003	0.988
H	-3.521	1.671	-0.653
H	-2.518	2.296	0.674
C	-0.770	0.781	-0.459
C	-1.250	-1.925	0.279
H	-3.254	-1.163	0.459
H	-2.646	-1.194	-1.202
N	0.105	-0.250	-0.835
O	-0.394	1.950	-0.573
C	0.055	-1.528	-0.339
H	-1.460	-2.959	0.005
H	-1.108	-1.885	1.363
H	1.011	0.069	-1.170
O	1.033	-2.260	-0.404
O	3.340	-0.526	-0.162
H	4.036	-0.714	-0.802
H	2.685	-1.231	-0.291
O	2.485	2.222	-0.298
H	1.519	2.202	-0.398
H	2.752	1.290	-0.231
N	1.035	0.176	2.107
H	1.335	0.359	3.061
H	1.200	1.034	1.587
H	1.684	-0.509	1.729

Zero-point correction	0.22662
Thermal correction to Energy	0.24422
Thermal correction to Enthalpy	0.24516
Thermal correction to Gibbs Free Energy	0.18185
Sum of electronic and zero-point Energies	-664.53228
Sum of electronic and thermal Energies	-664.51468
Sum of electronic and thermal Enthalpies	-664.51373
Sum of electronic and thermal Free Energies	-664.57705

Supplementary Table 6. Optimized geometries and energetics at the M06-2X/6-311++G level for the three-water concerted mechanism.**

R: cytidine

N	2.164	0.104	0.050
C	3.471	0.588	0.475
C	1.299	1.054	-0.546
H	3.354	1.368	1.228
H	3.998	1.016	-0.376
H	4.037	-0.247	0.885
C	1.758	-1.171	0.270
N	0.077	0.607	-0.966
O	1.675	2.205	-0.646
C	0.539	-1.607	-0.116
H	2.470	-1.815	0.773
C	-0.296	-0.649	-0.780
H	-1.378	1.680	-0.953
H	0.195	-2.612	0.076
N	-1.498	-1.035	-1.253
H	-2.121	-0.297	-1.555
H	-1.926	-1.847	-0.822
O	-2.330	1.822	-0.738
H	-2.540	2.738	-0.929
H	-2.484	0.859	0.811
O	-2.374	0.272	1.582
H	-1.683	0.676	2.111
H	-2.403	-1.530	1.345
O	-2.354	-2.461	1.054
H	-3.009	-2.939	1.565

Zero-point correction	0.20388
Thermal correction to Energy	0.22052
Thermal correction to Enthalpy	0.22146
Thermal correction to Gibbs Free Energy	0.15962
Sum of electronic and zero-point Energies	-663.30959
Sum of electronic and thermal Energies	-663.29296
Sum of electronic and thermal Enthalpies	-663.29201
Sum of electronic and thermal Free Energies	-663.35386

TS: cytidine

N	2.188	-0.058	0.084
C	3.519	0.345	0.526
C	1.256	0.959	-0.064
H	4.148	-0.541	0.582
H	3.459	0.819	1.505
H	3.946	1.060	-0.178
C	1.875	-1.366	-0.200
N	0.020	0.552	-0.535
O	1.518	2.114	0.173
C	0.645	-1.749	-0.569
H	2.689	-2.071	-0.089
C	-0.379	-0.738	-0.658
H	0.395	-2.781	-0.750
N	-1.543	-0.976	-1.283
H	-2.228	-0.225	-1.244
H	-1.936	-1.884	-1.074
H	-0.716	1.275	-0.560
O	-2.451	1.749	-0.578
H	-2.880	2.601	-0.659
H	-2.908	1.216	0.146
O	-3.273	-0.034	1.010
H	-3.910	-0.122	1.719
H	-2.341	-0.585	1.249
O	-1.181	-1.212	1.289
H	-0.675	-1.023	2.084

Zero-point correction	0.20073
Thermal correction to Energy	0.21564
Thermal correction to Enthalpy	0.21658
Thermal correction to Gibbs Free Energy	0.15899
Sum of electronic and zero-point Energies	-663.26157
Sum of electronic and thermal Energies	-663.24665
Sum of electronic and thermal Enthalpies	-663.24571
Sum of electronic and thermal Free Energies	-663.30330
$\Delta\Delta G$ Solvation	-1.8 kcal mol^{-1}

R: 5,6-dihydrocytidine

N	2.182	0.191	-0.068
C	3.413	0.673	0.534
C	1.686	-1.093	0.399
H	4.170	-0.110	0.468
H	3.270	0.946	1.587
H	3.751	1.554	-0.006
C	1.272	1.144	-0.489
C	0.688	-1.640	-0.603
H	2.533	-1.772	0.506
H	1.210	-0.995	1.387
N	-0.010	0.711	-0.862
O	1.554	2.322	-0.542
C	-0.296	-0.553	-0.937
H	0.159	-2.509	-0.209
H	1.197	-1.921	-1.530
H	-1.485	1.681	-0.700
N	-1.506	-0.941	-1.373
H	-2.199	-0.214	-1.513
H	-1.843	-1.849	-1.076
O	-2.450	1.756	-0.489
H	-2.707	2.674	-0.597
H	-2.571	0.637	0.923
O	-2.465	-0.037	1.622
H	-1.972	0.395	2.321
H	-2.243	-1.775	1.196
O	-2.056	-2.662	0.834
H	-2.592	-3.278	1.336

Zero-point correction	0.22745
Thermal correction to Energy	0.24449
Thermal correction to Enthalpy	0.24543
Thermal correction to Gibbs Free Energy	0.18267
Sum of electronic and zero-point Energies	-664.48841
Sum of electronic and thermal Energies	-664.47137
Sum of electronic and thermal Enthalpies	-664.47043
Sum of electronic and thermal Free Energies	-664.53319

TS: 5,6-dihydrocytidine

N	2.174	0.102	0.059
C	3.431	0.579	0.612
C	1.815	-1.279	0.365
H	4.234	-0.090	0.296
H	3.396	0.605	1.706
H	3.624	1.582	0.241
C	1.234	1.037	-0.261
C	0.714	-1.765	-0.558
H	2.708	-1.889	0.223
H	1.495	-1.374	1.406
N	-0.024	0.537	-0.661
O	1.417	2.232	-0.261
C	-0.378	-0.752	-0.667
H	0.295	-2.702	-0.197
H	1.106	-1.903	-1.572
N	-1.546	-1.094	-1.198
H	-2.250	-0.361	-1.289
H	-1.882	-2.013	-0.950
H	-0.766	1.239	-0.777
O	-2.639	1.496	-0.853
H	-3.110	2.316	-1.008
H	-2.909	1.124	0.054
O	-2.977	0.252	1.305
H	-3.766	-0.171	1.646
H	-2.105	-0.434	1.405
O	-1.027	-1.186	1.393
H	-0.565	-0.998	2.214

Zero-point correction	0.22515
Thermal correction to Energy	0.24018
Thermal correction to Enthalpy	0.24112
Thermal correction to Gibbs Free Energy	0.18338
Sum of electronic and zero-point Energies	-664.45305
Sum of electronic and thermal Energies	-664.43803
Sum of electronic and thermal Enthalpies	-664.43709
Sum of electronic and thermal Free Energies	-664.49483
$\Delta\Delta G$ Solvation	-2.1 kcal mol^{-1}
